# Surface Confined Hydrogenation of Graphene Nanoribbons

**DOI:** 10.1021/acsnano.1c11372

**Published:** 2022-07-05

**Authors:** Yi-Ying Sung, Harmina Vejayan, Christopher J. Baddeley, Neville V. Richardson, Federico Grillo, Renald Schaub

**Affiliations:** EaStCHEM and School of Chemistry, University of St Andrews, KY16 9ST, St Andrews, U.K.

**Keywords:** graphene, scanning tunneling microscopy, scanning tunneling spectroscopy, hydrogenation, nanoribbons

## Abstract

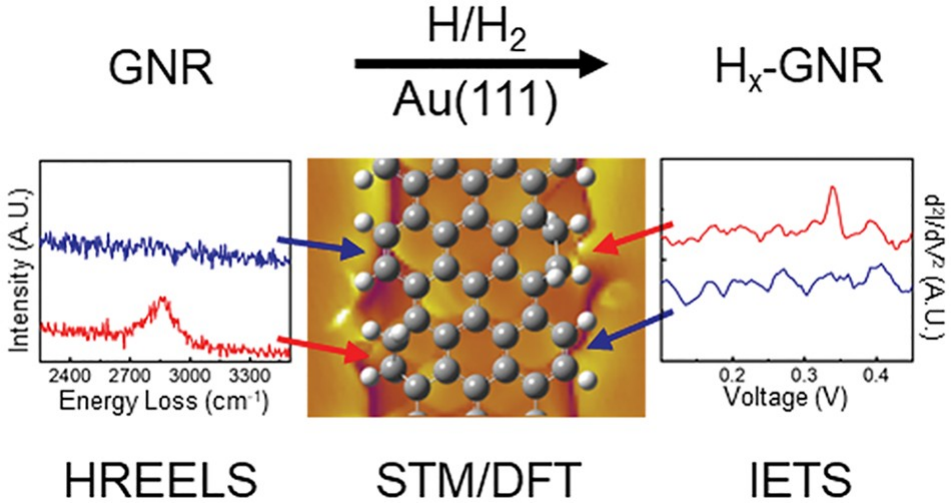

On-surface synthesis with designer precursor molecules is considered an effective method for preparing graphene nanoribbons (GNRs) of well-defined widths and with tunable electronic properties. Recent reports have shown that the band gap of ribbons doped with heteroatoms (such as boron, nitrogen, and sulfur) remains unchanged in magnitude in most cases. Nevertheless, theory predicts that a tunable band gap may be engineered by hydrogenation, but experimental evidence for this is so far lacking. Herein, surface-confined hydrogenation studies of 7-armchair graphene nanoribbons (7-AGNRs) grown on Au(111) surfaces, in an ultrahigh vacuum environment, are reported. GNRs are first prepared, then hydrogenated by exposure to activated hydrogen atoms. High resolution electron energy loss spectroscopy (HREELS) and scanning tunneling microscopy (STM) images reveal a self-limited hydrogenation process. By means of a combination of bond-resolved scanning tunneling microscopy (BRSTM) imaging and tip-induced site-specific dehydrogenation, the hydrogenation mechanism is studied in detail, and density-functional theory (DFT) calculation methods are used to complement the experimental findings. In all cases, the results demonstrate the successful modification of the electronic properties of the GNR/Au(111) system by edge and basal-plane hydrogenation, and a mechanism for the hydrogenation process is proposed.

As a two-dimensional material with outstanding electrical properties, graphene has potential in numerous applications.^1,2^ For example, graphene exhibits a large electron mobility (2 × 10^5^ cm^2^ V^–1^ s^–1^) at room temperature.^3^ However, pristine graphene is a semimetal, and therefore the absence of a band gap prohibits its use in digital nanoelectronic devices. Several strategies have proven to generate a finite gap through structural or electronic functionalization,^4,5^ including the etching of periodic structures to create antidot lattices,^6,7^ and the adsorption of hydrogen, which leads to structural distortions.^5^ Most interesting to the current study, a tunable and reliable band gap can be achieved through controlling the lateral extent of a graphene sheet, that is, by preparing graphene nanoribbons (GNRs).^8,9^ The controlled bandgap is achieved through edge effects and quantum confinement.^10^ Therefore, the magnitude of the band gap depends on the precise width and orientation of the GNRs.^4,11^ There are two types of edge structures: armchair and zigzag. For GNRs with armchair-shaped edges (referred to as AGNRs), each edge carbon atom is passivated by one hydrogen atom, and DFT calculations have shown that AGNRs can be either metallic or semiconducting depending on their widths.^8,9^ A bottom-up synthesis method for preparing atomically precise GNRs was proposed by the group of Roman Fasel in 2010.^12^ The appeal of this method is that it affords control over the width and edge geometry of the nanoribbons, both determined by the structure of the building blocks. The precursors can be designed to yield a broad range of GNRs of high structural precision and low defect density, in order to provide ultimate control over the bandgap. For example, an umbrella-shaped monomer is used to form 6-ZGNRs (with zigzag edges) with a resulting band gap of 1.5 eV.^13^

Heteroatom-doped GNRs can also be produced by this bottom-up approach, whereby dopant heteroatoms such as B or N are introduced with atomic precision either along the ribbon edges or within the carbon backbone.^14−18^ These site-specific doped GNRs possess electronic states introduced into the band gap, either by providing an empty orbital (B) or an electron lone pair (N and S).^19^ However, in most cases, the band gap of doped GNRs remains nearly unchanged in magnitude as compared to the pristine ribbons. Theoretical calculations explain that the modulation of the electronic properties strongly depends on the position of the dopants.^14,20^ Substitutional atoms within the backbone have a minimum effect on the electronic structure, while dopants at the edges have more of an effect. For example, backbone B-doped 7-AGNRs show only 0.1 eV reduction.^14^ Another example is N-doped chevron GNRs.^18^ Although the N dopants are incorporated at the apex of convex edges, the alteration of the electronic structure is comparatively minor. Sulfur offers electron lone-pairs that are in conjunction with the aromatic π-system and is used as dopant to prepare sulfur edge-doped 13-AGNRs.^21^ Due to the small difference in electronegativity between sulfur and carbon, the electronic properties remain relatively unchanged as compared to pristine 13-AGNRs.

The adsorption of hydrogen on graphene has been extensively studied due to its potential application in both electronic devices,^5,22−24^ and hydrogen storage.^25,26^ The hydrogenation process strongly depends on the binding between graphene and the substrate atoms on which it resides. For example, hydrogenation of graphene/SiC results in the formation of hydrogen dimer structures,^22^ which are also found on hydrogenated HOPG.^27,28^ In contrast, on transition metals such as Ir(111),^5^ and Pt(111),^29^ hydrogen displays an affinity for specific adsorption sites within the Moiré superstructure formed by the graphene layer and the substrate. However, all the results show a self-limiting process, indicating that graphene can only be partially hydrogenated up to a saturation coverage of 0.4 ML.^22,30,31^ To date, the hydrogenation reaction and its effects on the structural and electronic properties of GNRs have merely been studied through theoretical calculations.^32−35^ In this paper, we focus on the hydrogenation of 7-AGNRs prepared in ultra-high-vacuum (UHV) conditions on a Au(111) surface, accomplished by exposure to active hydrogen. High-resolution electron energy loss spectroscopy (HREELS) is used to prove a successful hydrogenation. Scanning tunneling microscopy (STM and Bond-Resolved STM) and spectroscopy (STS) data indicate that hydrogen interacts with GNRs to create short-range disorder that consequently modifies the electronic structure. Inelastic electron tunneling spectroscopy (IETS) demonstrates that both edge and basal-plane sites are hydrogenated. Finally, based on selective STM tip-induced dehydrogenation experiments, a step-by-step atomistic mechanism of hydrogenation is elucidated, and supported by density functional theory (DFT) calculations.

## Results and Discussion

**Figure 1:**
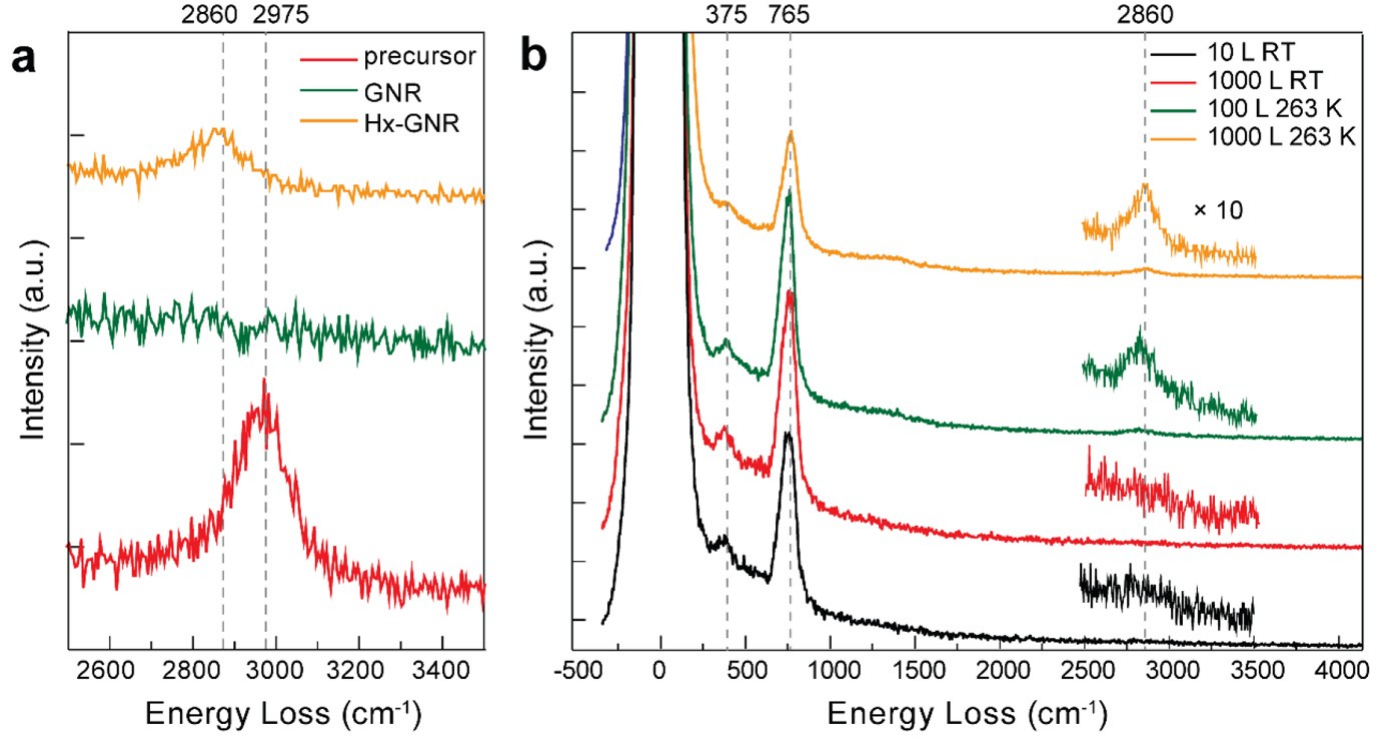
Comparison of HREEL spectra of 7-AGNRs obtained after different hydrogenation treatments on the Au(111) surface. (a) CH stretch energy window for DBBA precursors (red), pristine 7-AGNRs (green), and partially hydrogenated 7-AGNRs (yellow). (b) Full HREEL spectra of 7-AGNRs after exposure to atomic hydrogen at 300 K and 10 L (black); 300 K and 1000 L (red); 263 K and 100 L (green); 263 K and 1000 L (yellow). The CH stretch signal (from 2500 to 3500 cm^–1^) is magnified 10 times. Spectra are intensity offset for clarity.

To prepare 7-AGNRs, the precursor 10,10′-dibromo-9,9′-bianthracene (DBBA) was deposited onto a Au(111) surface, and then sequentially annealed to induce polymerization (475 K) and cyclodehydrogenation (675 K).^12^ Representative HREEL spectra following the preparation of the 7-AGNRs, focusing on the diagnostic CH stretch region, are shown in Figure 1a. Full scale energy loss spectra are reported in Figure S1 alongside a comparison to gas phase calculated spectra in Figure S2. The vibrational spectra of DBBA after deposition on the Au(111) surface (Figure S1, red traces) and that of the polymers produced upon the first annealing step (Figure S1, blue traces) are very similar. This indicates that when polymerization occurs, the dihedral angle between consecutive anthracene units remains essentially unchanged. In the spectrum recorded after inducing cyclodehydrogenation to form AGNRs (Figure S1, green traces), all molecular vibrations show a decrease in intensity, except the out-of-plane bending mode, δ(CH)oop, at 765 cm^–1^. The AGNRs lie essentially flat on Au(111) and, as a consequence, in-plane modes become only weakly dipole active since their inherent dipole moments are parallel to the metal surface. For example, the aromatic CH stretch mode (2975 cm^–1^) observed in the spectra of both DBBA and polymer is not present in the spectrum of the AGNRs. Upon exposure to active hydrogen at a specific temperature (263 K, discussed further below), a peak at 2860 cm^–1^ is observed (Figure 1a, yellow trace). This peak is assigned to an aliphatic out-of-phase CH_2_ stretch mode, indicating that a change of hybridization of carbon atoms from *sp*^2^ to *sp*^3^ has occurred. We note here that exposure to unactivated H_2_ (from 80 to 300 K, and up to 1000 L) leads to no evidence of hydrogenation as observed from HREELS and STM data.

HREELS measurements were also performed in order to optimize the hydrogenation conditions. Figure 1b shows four HREELS measurements recorded on 7-AGNRs after different hydrogenation conditions. In contrast to the room temperature data (black and red traces), the spectra recorded after hydrogenation at 263 K (green and yellow traces) show a broad peak at 2860 cm^–1^, revealing that a lower temperature is required for the hydrogenation process. A lower temperature increases the residence time of the hydrogen atoms to promote their reaction with the GNRs. In addition, the spectrum corresponding to an increased dose of H/H_2_ (yellow trace) shows only a marginal increase in intensity at 2860 cm^–1^, while the peak at 765 cm^–1^ decreases in intensity, as compared to the green trace. These minor changes in magnitude of the vibrational modes upon increased exposure signify that the extent of hydrogenation is limited, as will be later shown by STM measurements.

**Figure 2:**
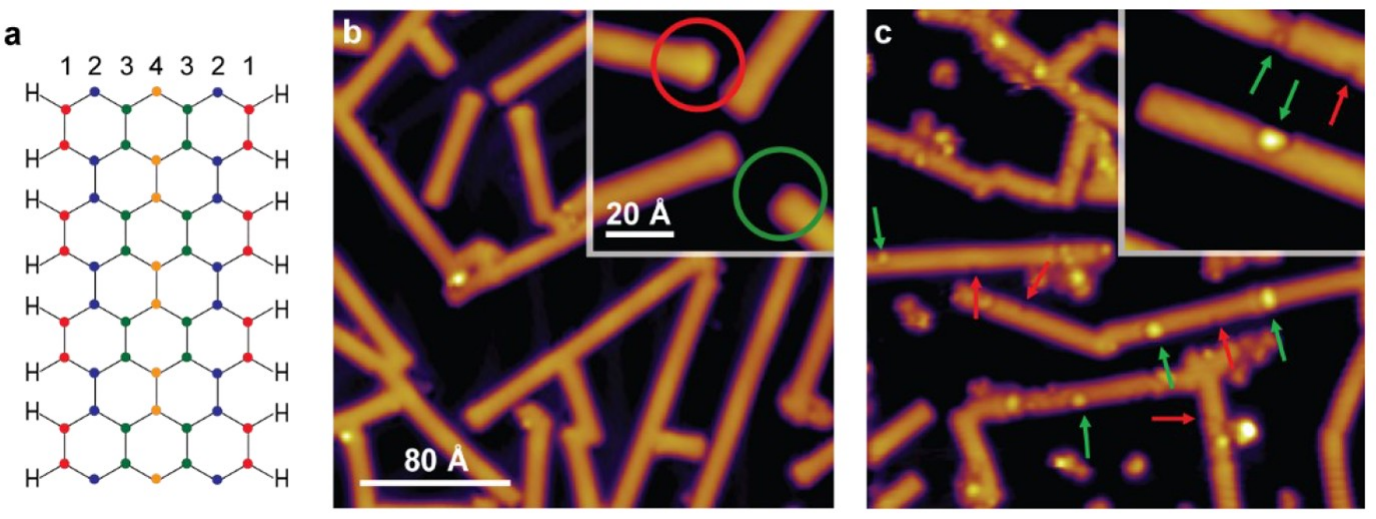
Comparison of STM images of pristine and hydrogenated 7-AGNRs. (a) The structure of pristine 7-AGNR with the carbon atoms indexed for reference. C1 refers to armchair edge carbon atoms, while C2, C3, C4 refer to basal plane carbon atoms. (b) Before and (c) after hydrogenation at 223 K. Two features, edge defects and protrusions, are marked by red and green arrows, respectively. Two GNR termini that differ in appearance are highlighted in the inset of (b) by red and green circles (see Section SI2 for BRSTM images of the two termini). Tunneling parameters: (b) *V* = 1.08 V, *I* = 0.1 nA; (c) *V* = 0.08 V, *I* = 0.8 nA.

Ahead of reporting our microscopy data, Figure 2a shows the structure of a 7-AGNRs in which the various carbon atoms are indexed for clarity in subsequent discussions. Figures 2b and 2c show STM images acquired before and after exposure to active hydrogen at 223 K, respectively. The GNRs are readily recognizable, with noticeable differences appearing as a direct result of the hydrogenation. Prior to the exposure, the defect-free ribbons exhibit two termini that differ in appearance. Their chemical identity is well documented:^36,37^ an enlarged terminus (red circle in inset) is associated with a monohydrogenated zigzag edge (*sp*^2^ character) and a featureless terminus (green circle) to a dihydrogenated zigzag edge (*sp*^3^). Bond-resolved STM images of both termini are reported in Section SI2 (Figure S3). By comparison, hydrogenated ribbons exhibit only featureless termini. Hence, this transformation represents a first microscopy evidence of successful GNR hydrogenation. Further evidence for potential reaction sites can be found in the form of emerging features on the AGNR sections, of which we observe two types: (1) edge defects (marked with red arrows in Figure 2c) that appear as indentations only along the edges of the AGNRs, and (2) brighter protrusions (green arrows) that tend to locate either at one side and extend into the nanoribbon or cover the entire width of the ribbon. While edge defects are of a similar size and shape, protrusions tend to be much larger and irregular in shape.

**Figure 3:**
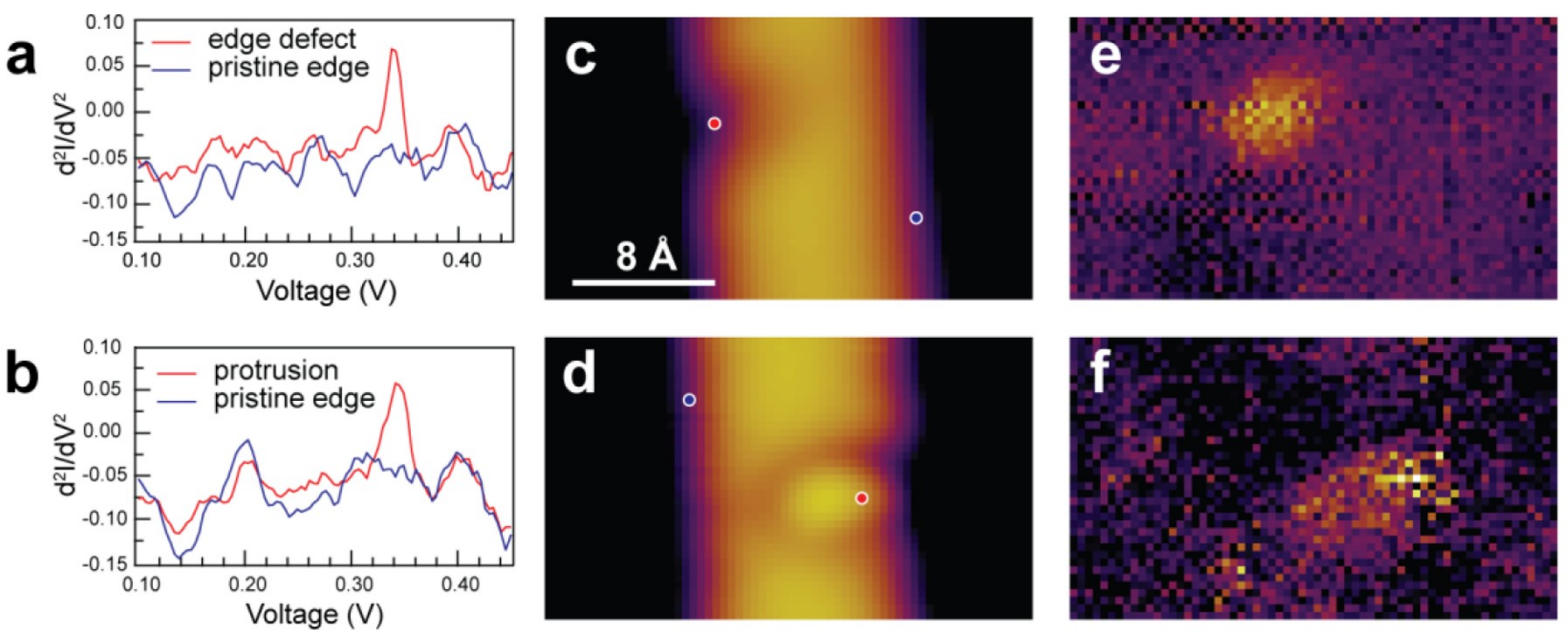
d^2^*I*/d*V*^2^ spectra of hydrogenated features, and corresponding STM and IETS imaging. Single point d^2^*I*/d*V*^2^ spectra recorded at (a) an edge defect and (b) a protrusion imaged, as shown in (c,d). Standard STM images of (c) the edge defect and (d) the protrusion. The colored dots indicate typical locations of d^2^*I*/d*V*^2^ spectra acquisition. d^2^*I*/d*V*^2^ maps of (e) the edge defect and (f) the protrusion seen in (c,d). STM images and d^2^*I*/d*V*^2^ maps are acquired simultaneously on the same features shown in (c,d). Tunneling and spectroscopy parameters: *V* = 340 mV, *I* = 1 nA, *f* = 632 Hz, *V*_ac_ = 20 mV.

To confirm that both features are indeed reaction sites, we employ d^2^*I*/d*V*^2^ spectroscopy and imaging. Figures 3a and 3b (red traces) show single-point d^2^*I*/d*V*^2^ spectra acquired over two features identified as edge defect and protrusion, respectively, and two such features are shown in Figures 3c and 3d, respectively. For reference, d^2^*I*/d*V*^2^ spectra acquired on pristine ribbon edges near the features are also reported (blue traces). The colored dots in Figures 3c and 3d indicate typical locations at which we acquire such d^2^*I*/d*V*^2^ spectra. The two d^2^*I*/d*V*^2^ spectra show a sharp resonance at 341 mV (ca. 2750 cm^–1^), which is only present when acquired over the features. The intensity of the resonance varies slightly with different STM tips and with the position of the tip over the features, but its energy remains consistently near 341 mV. Hence, this resonance can be identified as the stretch mode of a C–H bond, in line with previously reported C–H bond stretch mode measurements (359 mV, ca. 2895 cm^–1^) acquired on an acetylene molecule adsorbed on the Cu(100) surface.^38^ This value is also in good agreement with the energy loss features measured in HREELS (Figure 1b). The downward energy shift of the observed resonance is expected due to the different carbon hybridizations. Indeed, acetylene has *sp* hybridization, while the hydrogenated nanoribbons have *sp*^*3*^ hybridization. The dipole moment of this *sp*^*3*^ C–H stretching mode possesses a normal component with respect to the surface, as does the *sp* C–H stretching mode of acetylene, for which the surface dynamic dipole is enhanced due to both hydrogen atoms moving away from the Cu surface and so can be excited in an inelastic electron tunneling (IET) process.^38^ In contrast, the *sp*^*2*^ C–H bonds along the edges of the pristine AGNRs are nearly parallel to the Au(111) surface, resulting in the absence of an associated IET resonance. We note that the two d^2^*I*/d*V*^2^ spectra reported in Figure 3b exhibit a resonance at 0.2 V, while it is not observed in Figure 3a. Without consideration of STM tip-artifacts, the two blue traces (both spectra acquired on pristine edges) should display the same resonances. We therefore conclude that the 0.2 V feature seen in Figure 3b arises from the electronic structure of the STM tip used for data acquisition—a different tip from that used for acquisition of the spectra in Figure 3a. Further support for this assertion stems from the absence of any corresponding resonance in our HREELS data (expected at 1600 cm^–1^, see Figure 1b).

Constant current d^2^*I*/d*V*^2^ imaging at a bias set near to the value of the resonance (341 mV) is utilized to map the spatial distribution of the C–H stretch intensity across the two distinct features. As can be seen in Figures 3e and 3f, a signal is observed only at the locations of the features. In contrast, a signal is neither observed on the rest of the nanoribbon nor on the Au(111) surface. Hence, the C–H stretch signal only exists in direct correspondence to the topographic features, further confirming that hydrogenation has occurred on both edge and basal-plane sites. However, the resolution achieved in all standard STM topography measurements (as shown in Figures 2c, 3a, and 3b) is just not sufficient to identify the exact sites of hydrogen attack. Therefore, bond-resolved STM (BRSTM) imaging is used to explore the intramolecular structure of 7-AGNRs.

**Figure 4:**
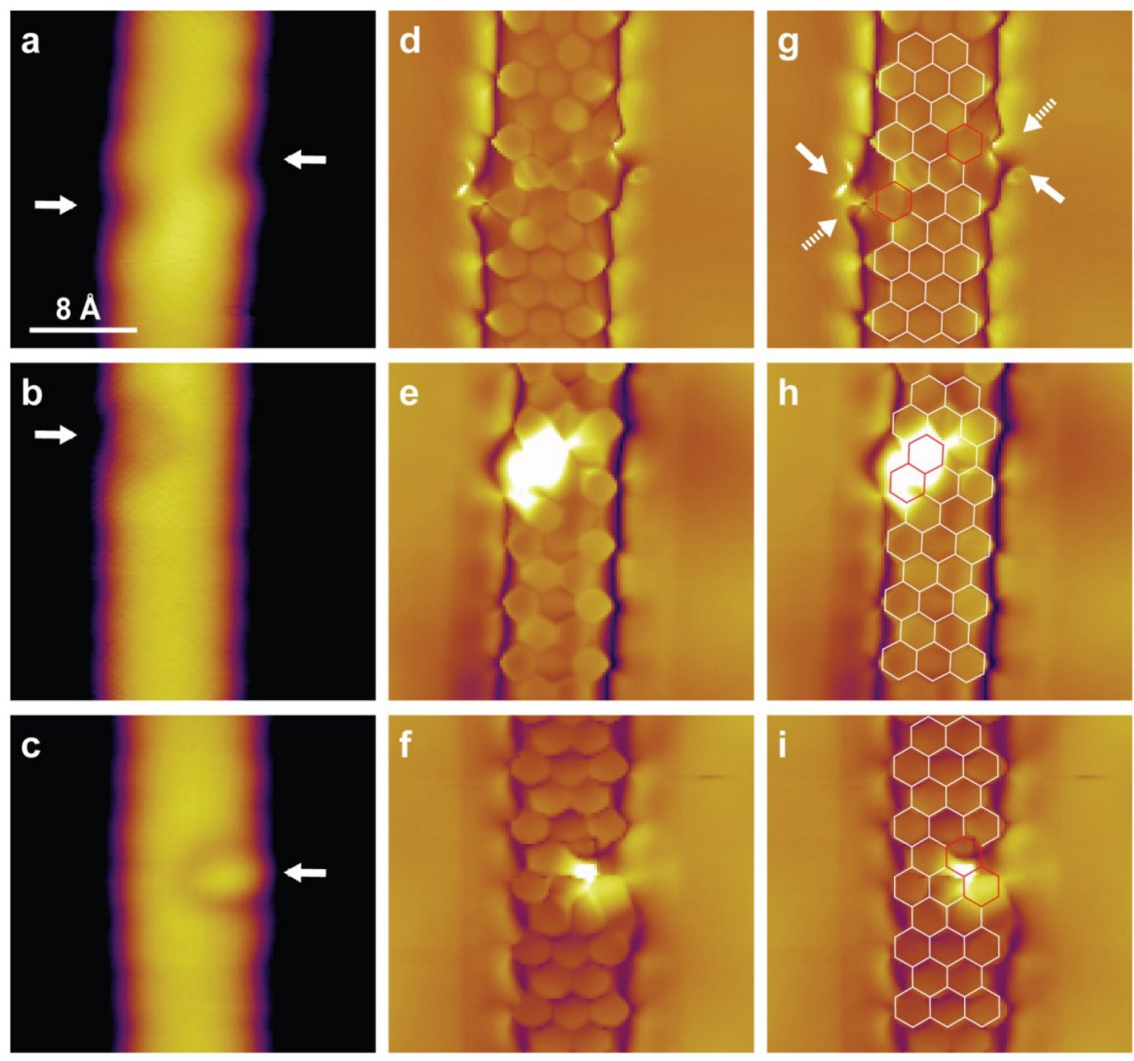
STM and BRSTM images of a small edge defect, a large edge defect, and a small protrusion. The STM topographic images with a CO-functionalized tip on the hydrogenated 7-AGNRs show (a) two small edge defects marked by arrows, (b) a large edge defect, and (c) a small protrusion. Panels (d), (e), and (f) are corresponding BRSTM images. The superposed models in (g), (h), and (i) highlight the perturbed electronic contrast arising from hydrogen addition. Tunneling parameters: (a) *V* = 1 V, *I* = 1 nA; (b) *V* = 1 V, *I* = 0.5 nA; (c) *V* = 1 V, *I* = 0.1 nA; (d) *V* = 80 mV, *I* = 0.7 nA, *f* = 427 Hz, *V*_ac_ = 60 mV; (e) *V* = 40 mV, *I* = 1 nA, *f* = 427 Hz, *V*_ac_ = 60 mV; (f) *V* = 40 mV, *I* = 1 nA, *f* = 427 Hz, *V*_ac_ = 60 mV.

Figure 4 reports standard STM images (Figure 4a–c), BRSTM images (Figure 4d–f), and superposed models (Figure 4g–i) of three hydrogenated features. The first two sets of data are acquired on two edge defects that are revealed to be structurally different only thanks to the BRSTM data. Henceforth, we label these two different edge defects as small (Figure 4a,d,g) and large (Figure 4b,e,h) edge defects. The third set is acquired on a protrusion possessing the smallest identified size (Figure 4c,f,i). The BRSTM image in Figure 4d, corresponding to the standard topography in Figure 4a, shows two small edge defects highlighted with arrows on each side of a GNR section. The resolution of the BRSTM image is sufficient to differentiate three types of hexagonal C_6_ rings from their topographic appearance. First, inner (basal-plane) rings are easily recognizable from their nearly regular 6-fold symmetry. Second, pristine edge rings show only a subtle deviation from the expected hexagonal structure arising from the termination of the edge carbon atoms each with one hydrogen atom (*sp*^2^ configuration, see Figure 2a). And third, distorted edge rings are observed at the two locations where indentations can be identified in the standard STM image. With these three differences in mind, and thanks to the fact that the structural contrast is sufficient to identify the −3–2– alternation of anthracene units along the ribbon, a superposed model is built, revealing the edge C_6_ rings directly affected by the hydrogenation (Figure 4g, red hexagons). Upon closer inspection, the defect on the left-hand side of Figures 4d and 4g displays (where we anticipate the peripheral hydrogen atoms to be) a higher electronic contrast at the top of the affected C_6_ ring (solid arrow), and a lower contrast at the bottom (dashed arrow), while the feature on the right side displays a lower contrast at the top of the affected C_6_ ring (dashed arrow) and a higher contrast at the bottom (solid arrow). This subtle asymmetric appearance (made more obvious in Figure S4 by image filtering) indicates that the edges of the GNR affected by hydrogenation are slightly distorted (deviating from planarity) as a direct consequence of the change of edge carbon configuration from *sp*^2^ to *sp*^3^. Thus, the apparent small edge defects can be assigned to hydrogenation solely involving ribbon edge sites (C1 sites from Figure 2a). Whether the defects relate to the hydrogenation of a single C1 site, or both C1 sites constituting the affected C_6_ edge ring, will be discussed later.

The case of a large edge defect is presented in Figures 4b and 4e. Although the appearance of the large edge defect is rather similar to that of the small edge defect in topography, the BRSTM data display marked differences, with a more distorted structure for the large edge defect. In fact, BRSTM is well-known to be ideally suited to investigate planar aromatic systems, but less so for distorted systems. As can be seen in Figure 4e, the image loses resolution at the location of the defect. Nevertheless, a structural model is superposed on the BRSTM image to illustrate that the composition of this feature involves two adjacent C_6_ rings, including one edge ring and one inner ring, both highlighted in red (Figure 4h). Besides the hydrogenation of edge sites, as for the small edge defects discussed above, the involvement of the inner C_6_ ring indicates the occurrence of hydrogenation on the basal plane. Although the exact hydrogenated sites cannot be discerned from the images, it is reasonable to assume basal plane sites are indeed affected (we will later demonstrate these correspond to C2 sites from Figure 2a).

Figures 4c and 4f report the case of a small protrusion. The lack of bond resolution at the location of the feature is a clear indication of a significant loss of planarity upon hydrogenation. The extent of the disrupted structural contrast suggests the involvement of two adjacent hexagonal rings (marked in red in the superposed model), analogous to the large edge defect. Upon closer inspection, the small protrusion shows more extension toward the inner portion of the GNR as compared to both edge defects. This indicates that the hydrogenation, although involving the two same C_6_ rings as for the large edge defect, must affect different carbon atoms (we will later demonstrate these correspond to C3 region from Figure 2a).

**Figure 5:**
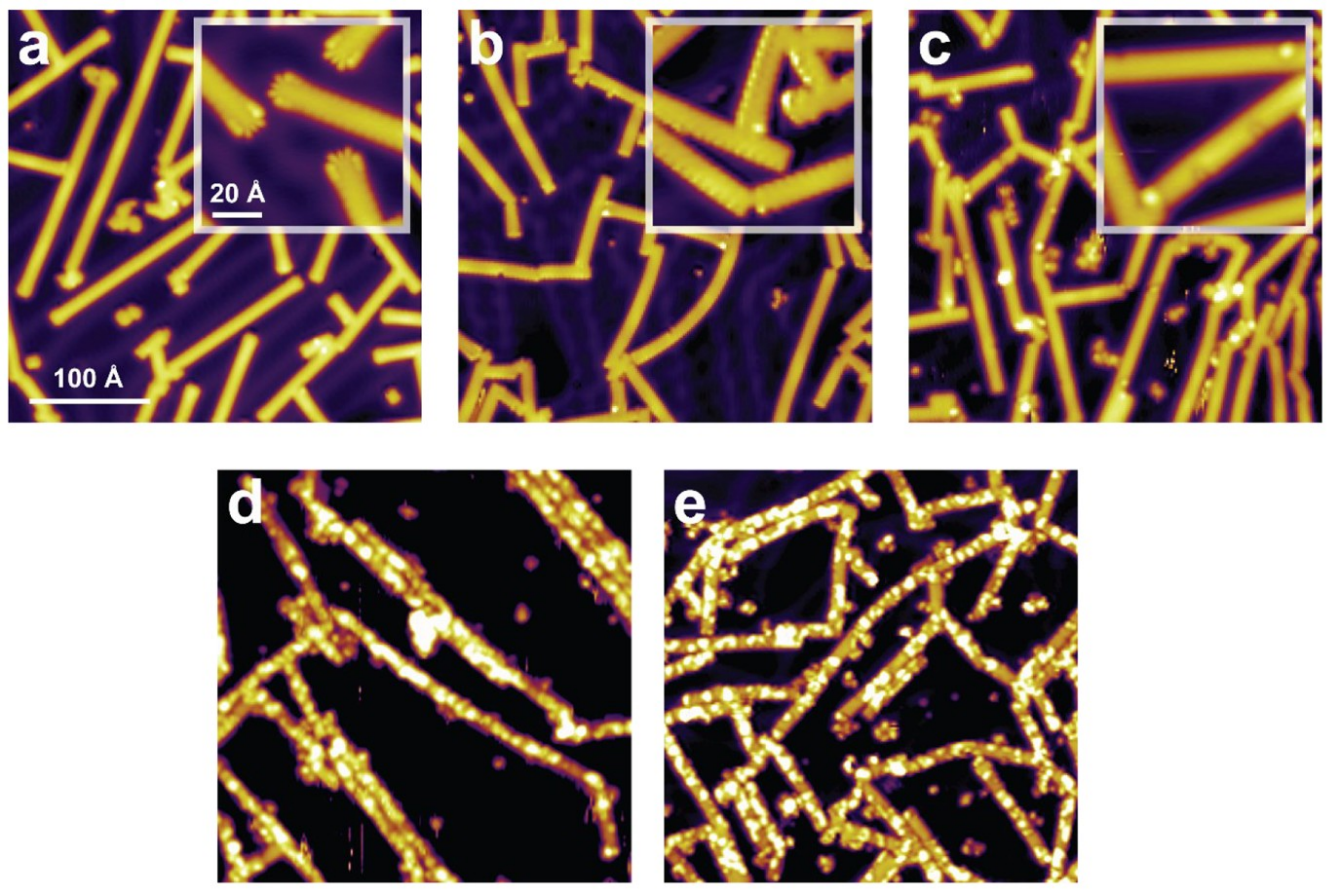
Hydrogenation as a function of hydrogen exposure. (a) Pristine 7-AGNRs on the Cu(111) surface. 7-AGNRs exposed to atomic hydrogen for (b) very low dose, (c) 200 s at 1 × 10^–6^ mbar, (d) 300 s at 2 × 10^–6^ mbar, and (e) 900 s at 5 × 10^–6^ mbar. The insets in (a) and (b) show the change in the termini structure from CH to CH_2_ upon hydrogenation. The inset in (c) shows defects appearing along the edges. Tunneling parameters: (a) *V* = 1.0 V, *I* = 0.06 nA (inset *V* = 0.02 V, *I* = 1.0 nA); (b) *V* = 0.06 V, *I* = 0.79 nA (inset *V* = 0.05 V, *I* = 0.72 nA); (c–e) *V* = 1.0 V, *I* = 0.1 nA.

The identification of different hydrogenation sites with either different hydrogen content or geometric arrangements, and in particular the observation that all defects are connected to the ribbon edges, suggests a reaction mechanism that first involves hydrogenation attack of the outmost carbon atoms (edges), followed by the incremental hydrogenation of neighboring basal-plane carbon atoms. To assess this mechanism, STM measurements are performed to monitor the exposure-dependent evolution of the hydrogenation features, see Figure 5. First, in the absence of H/H_2_ exposure (Figure 5a), defect-free ribbons appear as expected, with either enlarged (CH) or featureless (CH_2_) termini. After exposure to a very low dose of active hydrogen (Figure 5b), defect-free ribbons with solely featureless (CH_2_) termini are observed (compare the insets in Figures 5a and 5b). As discussed earlier, this signifies the very onset of ribbon hydrogenation. For the intermediate exposure of 200 s at 1 × 10^–6^ mbar in Figure 5c, mostly indentation defects emerge along the edges (with a minority of protrusions also observed). When the exposure is increased to 300 s at 2 × 10^–6^ mbar, the STM image in Figure 5d reveals that protrusions become the dominant defects. These merge and form elongated structures covering the entire width of the ribbons, but not their lengths. Since the ribbons are only partially hydrogenated rather than fully hydrogenated, we have attempted to further increase the dose of activated hydrogen. As can be seen in Figure 5e (5 × 10^–6^ mbar, 900 s), the protrusions cover the entire width of the ribbons, but are seemingly prevented to further extend along the ribbon length. Thus, we do not observe any significant quantitative differences in the number of protrusions compared to Figure 5d. In the attempt to fully hydrogenate the nanoribbons, the H/H_2_ dose was further increased, but this did not lead to any appreciable topographic changes. This confirms that the hydrogenation mechanism is self-limited, in agreement with our HREELS data.

The amount of adsorbed hydrogen is very difficult to quantify for two main reasons: our STM data do not have sufficiently high resolution to allow for the hydrogen coverage to be quantified, and as a consequence, we do not know the packing structure that the hydrogen adopts within the bright and elongated protrusions. Nevertheless, with some assumptions of packing density and further image analysis (detailed in Section SI4), we estimate a saturation coverage of just under 40%, comparable to the hydrogenation of graphite and graphene.^22,30,31^

Our coverage-dependent STM data suggest the following: (1) Active hydrogen first attacks ribbon termini; (2) This is followed by hydrogenation of ribbon edges forming small edge defects (C1 region), which remain constrained in lateral extension; (3) Only then can basal plane sites chemisorb hydrogen, and this can proceed via two distinct pathways involving either one of the two basal-plane C2 or C3 regions. Extension to the C2 region is associated with a large edge defect in our topography, while extension to the C3 region corresponds to a small protrusion. Experimental evidence for hydrogenation extending further onto the basal plane is confirmed by BRSTM imaging resolving larger protrusions (see Section SI5, Figure S6).

**Figure 6:**
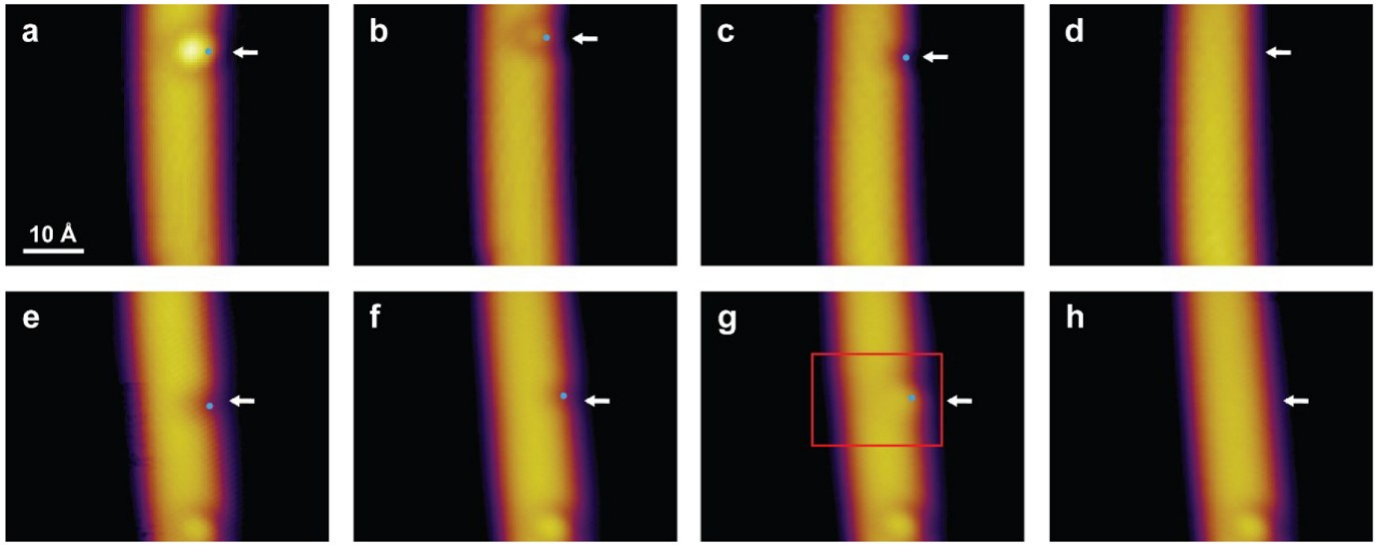
Stepwise STM tip-induced dehydrogenation. Example 1: STM image of (a) a large protrusion, (b) a small protrusion, (c) a small edge defect, and (d) a pristine nanoribbon. Example 2: STM image of (e) a large edge defect, (f) a small edge defect, (g) an intermediate feature, and (h) a featureless site. The blue dots highlight the exact sites where voltage pulses are applied. Tunneling parameters (a–d): *V* = 0.5 V, *I* = 0.03 nA; (e–h): *V* = 0.5 V, *I* = 0.1 nA.

To provide experimental validation of our proposed hydrogenation mechanism, and hence the sequence of incremental H additions, we proceed with experiments whereby the STM tip is employed to electron-stimulate the desorption of H atoms by applying a voltage pulse over specific defects. BRSTM and STM imaging are then utilized to resolve the changes in intramolecular structure of 7-AGNRs to identify the sites of hydrogen desorption, and therefore (in reverse) of adsorption. Two examples are reported in Figure 6. The first case of stimulated desorption starts from a large protrusion (arrow in Figure 6a), and as a consequence of a series of applied voltage pulses, the protrusion is revealed to sequentially transform in appearance into a small protrusion (Figure 6b), a small edge defect (Figure 6c), and finally a pristine nanoribbon (Figure 6d). The second example shows the manipulation of a large edge defect (arrow in Figure 6e) evolving into a small edge defect (Figure 6f), a yet unobserved asymmetric feature (Figure 6g), and finally a pristine nanoribbon (Figure 6h).

There are two important observations to draw from the electron-stimulated desorption results. First, these allow for a hierarchical sequence in hydrogen content to be recognized among the structures observed, and second, a yet-unobserved asymmetric feature is identified only as a direct consequence of the STM tip excitation. We have never observed this feature after sample preparation (the BRSTM imaging used to resolve this feature is reported in Section SI6, Figure S7). Further manipulation of this feature shows that it can be dehydrogenated into a pristine 7-AGNR. Another example of an asymmetric feature is reported in Section SI6, Figure S8. This feature is observed after the manipulation of a large edge defect when converted to a small edge defect. Since both intermediate asymmetric features arise from STM manipulation, these can be assigned to configurations that are only artificially stabilized at 5 K (the STM acquisition temperature). Therefore, this suggests these intermediate species can be associated with odd numbers of adsorbed hydrogen atoms. Accordingly, we propose that hydrogenation proceeds via a pairwise addition mechanism: a first hydrogen atom adsorbs at an edge carbon atom leading to the local loss of π character between the hydrogenated carbon atom and the adjacent one; this renders the adjacent carbon atom receptive to further hydrogen attack.

**Figure 7:**
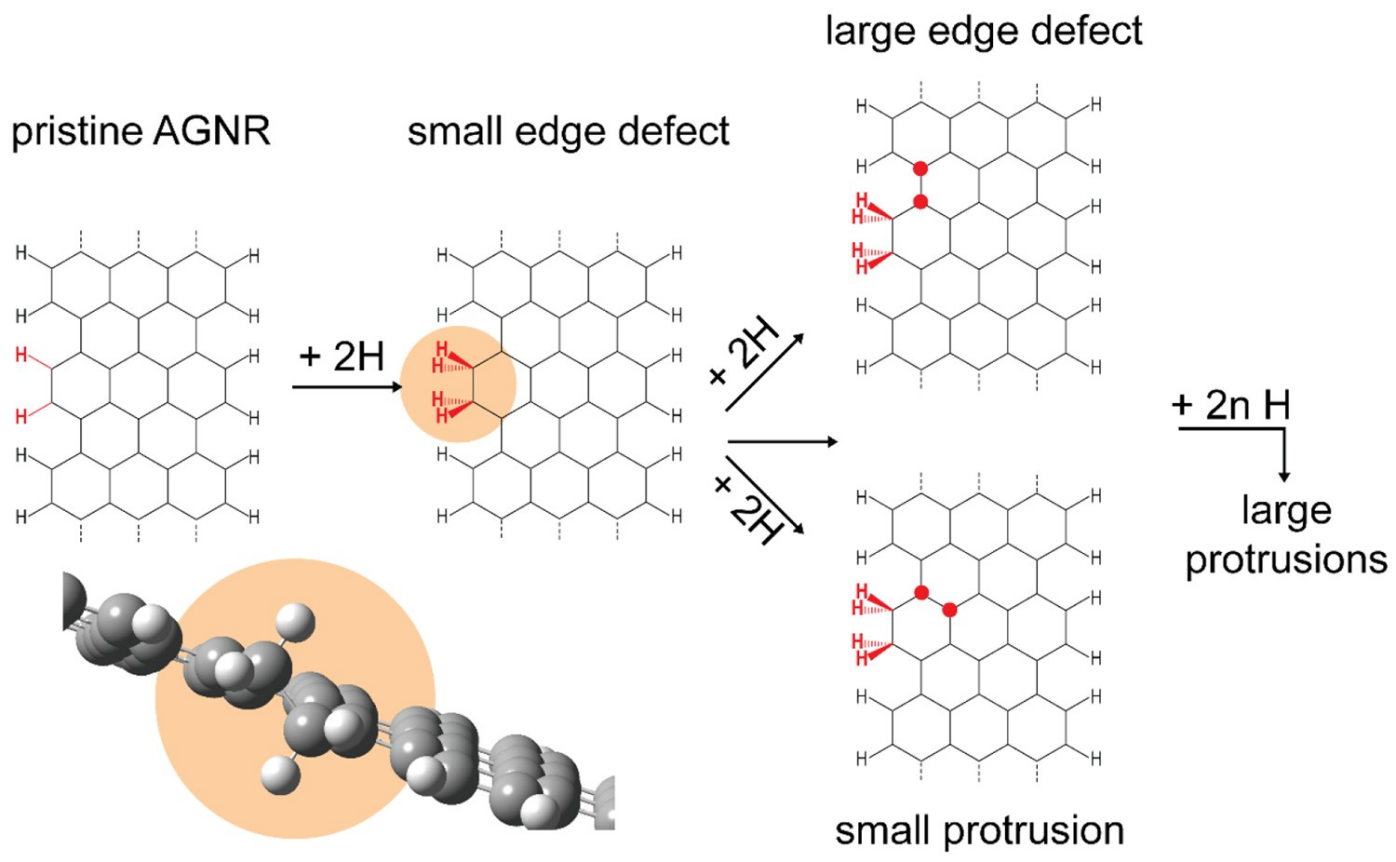
Schematic representation of the initial stages of hydrogenation of 7-AGNRs on Au(111). The molecular ball model displayed on the bottom-left of the panel serves to illustrate the distortion we anticipate being present at the GNR edges as a result of hydrogenation.

From over 20 stimulated desorption experiments similar to those in Figure 6, we consistently observe that both small protrusions and large edge defects transform into small edge defects. We have never observed the interconversion between a large edge defect and a small protrusion. This suggests that, subsequent to the pairwise addition of two H atoms at one edge C_6_ ring (C1 region), further pairwise hydrogenation can follow two competing reaction pathways restricted to the basal plane: involving solely the C2 region (giving rise to a large edge defect) or the C2 and C3 region (a small protrusion). There on, increased hydrogenation leads to basal plane sites adsorbing further amounts of H atoms via the pairwise addition mechanism, giving rise to protrusions of varied sizes and shapes. The following key facts are noticeable: (1) Every observed hydrogenated feature (protrusion or edge defect) is in direct contact with a nanoribbon edge, confirming that hydrogenation must first proceed at edge carbon atoms. (2) The hydrogenation follows a pairwise addition mechanism as evidenced by the observation of intermediate configurations in tip-assisted desorption data. (3) Further hydrogenation is only possible on basal plane sites near pre-existing hydrogenated sites. And finally, (4) the extent of hydrogenation is self-limited. Our hydrogenation mechanism of 7-AGNRs supported on a Au(111) surface is schematically summarized in Figure 7.

Previous DFT calculations by Choe *et al*.^34^ support our observations of a reaction onset being energetically favorable at ribbon edges. Their results show that the first edge-adsorbed hydrogen has the lowest formation energy (∼1.35 eV). Then the second hydrogen adsorbs preferentially to the adjacent carbon site of a C–C pair due to having the second lowest energy configuration (∼0.2 eV). The relaxation of the geometric strain induced by rehybridization results in a buckled C–C pair—a distortion also evidenced in our STM data and illustrated by the ball model in Figure 7. Our experimental results also indicate that once an edge defect is formed, further reaction proceeds onto the basal plane (in direct vicinity of the hydrogenated edge), while it is limited in extent along the length of the ribbon. These observations are not supported by previous DFT calculations.^33,34^ The authors conclude that further hydrogen addition will occur only at the edges until all the edges are hydrogenated. We ascribe the discrepancy to limitations in the theoretical model systems used in their work, arising from the choice of the periodic boundary conditions. We therefore performed DFT calculations on two different model systems that consist of the following: (1) A finite 7-AGNR composed of 10 anthracene units with CH_2_ termini and with a hydrogenated C–C pair at the sixth anthracene unit. (2) An infinite ribbon with a hydrogenated C–C pair repeated after 4 unit cells. This separation is sufficient to minimize the physical interaction between adjacent hydrogenated C–C pairs. The detailed results of our calculations are reported in Section SI7, and in what follows, we summarize our key findings. Our starting configurations agree in all aspects with the previous DFT calculations with a single tilted edge C–C pair (small edge defect observed in our STM images) favored over a flat edge configuration (Table S2). From there on, it is energetically more favorable by approximately 0.26 eV to hydrogenate a basal-plane carbon neighboring the edge pair (C2 region) than for the reaction to proceed along the ribbon edge (Table S3). Since our microscopy data indicate that the hydrogenation involves the pairwise addition of hydrogen atoms, simple geometric arguments show that a fourth hydrogen can then accommodate on two inequivalent basal plane sites, giving rise to either a large edge defect (C2) or a small protrusion (C3) as observed in our STM images.

**Figure 8:**
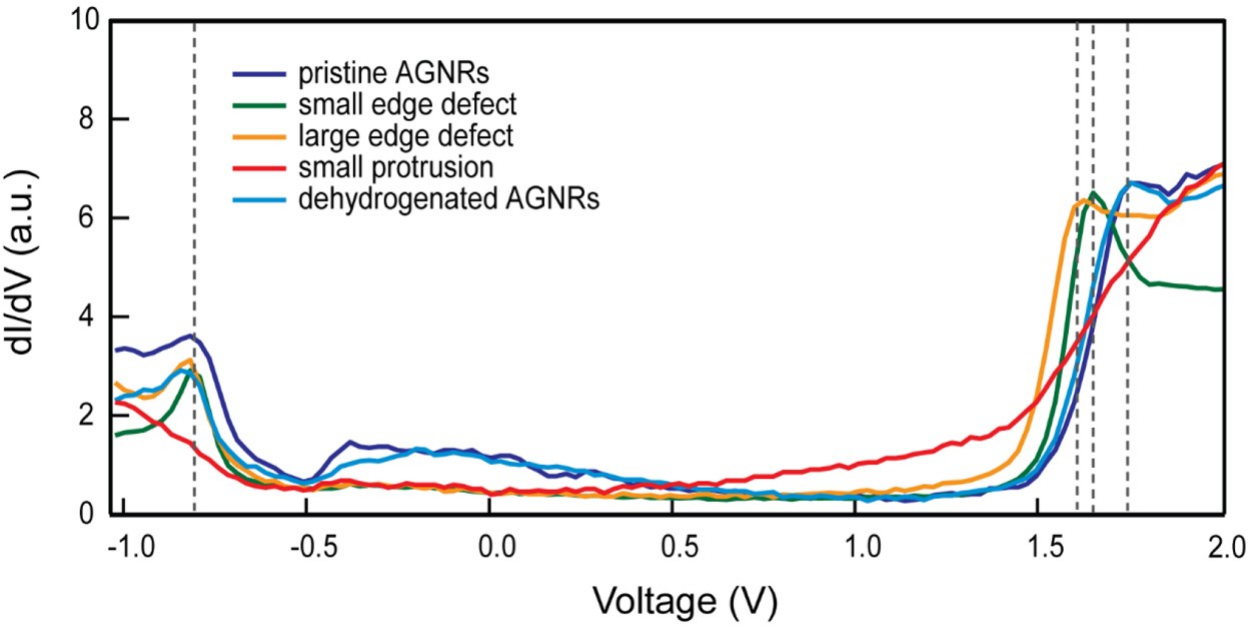
STM d*I*/d*V* point spectra acquired at the pristine AGNR (blue), small edge defect (green), large edge defect (yellow), small protrusion (red), and dehydrogenated AGNR (light blue).

In order to study the effect of hydrogenation on the electronic properties of the 7-AGNRs, the local electronic structure is probed using constant-height d*I*/d*V* single point spectroscopy as shown in Figure 8. The blue and light-blue traces are acquired on the edges of pristine AGNRs, prior to hydrogenation and after the complete STM-tip dehydrogenation of the various features we have identified (as performed in Figure 6), respectively. Both spectra are very similar, displaying well resolved valence edges (VBE, −0.84 eV) and conduction band edges (CBE, 1.77 eV). The resulting band gap for both pristine ribbon edges is 2.61 eV (with a standard deviation of 0.05 eV over approximately 20 recorded spectra), in fair agreement with previous experimental results (2.7 eV).^37,39^ This indicates that the hydrogenated ribbons can be reverted to their original defect-free state via tip-assisted dehydrogenation. We note the presence in both spectra of the broad signal near the Fermi level (from −0.66 to 0.68 eV) associated with the Au(111) surface state. A downward shift of the CBE is observed for the spectra recorded on both small edge defects (green trace, 1.64 ± 0.02 eV) and large edge defects (yellow trace, 1.61 ± 0.02 eV), whereas the VBE remains essentially unperturbed as compared to pristine 7-AGNRs. For the latter, we observe the emergence of electronic states within the band gap. The spectra recorded on small protrusions (red trace) are markedly different, making it difficult to identify confidently the VBE and CBE. Nevertheless, a significant increase in LDOS becomes apparent near the Fermi level, pointing toward a decrease of the local band gap with the emergence of a metallic character imparted to the hydrogenated ribbons.

The downshift of CBE induced by edge defects indicates that the local electronic properties of the nanoribbons are modified upon hydrogenation, and in particular, that the local band gap is reduced. Our observations are quite at odds with simple arguments based on edge effects and quantum confinement, as well as DFT calculations.^34^ Hydrogenation is expected to reduce the aromaticity of the ribbons and increase their aliphatic character. Consequently, the predictions are that the hydrogenated AGNRs must exhibit an increased band gap. However, this applies only to gas-phase GNRs. The effect of the substrate is not considered. We suggest that the decrease of the local band gap is caused by electronic interaction between ribbons and the Au(111) substrate as a direct result of the structural distortions introduced by rehybridization of carbon atoms.

## Conclusion

We have shown through a combination of microscopy and spectroscopy the successful hydrogenation of 7-AGNRs supported on Au(111). The hydrogenation reaction is only enabled by exposure to active hydrogen. We have identified several hydrogenation features involving both edges and basal-plane sites. Through H/H_2_ exposure dependent studies, we demonstrated that the reaction first starts at ribbon terminations, then proceeds at ribbon edges and, from there on, extends onto the basal plane of the ribbons. The reaction is inhibited in extension along the ribbon length, indicating a self-limited hydrogenation process. By combining STM tip-induced hydrogen desorption measurements with DFT calculations, we proposed a mechanistic sequence of hydrogen pairwise addition reactions initiated at armchair edges, followed by two competing pathways extending onto the basal-plane of the ribbons. Finally, local electronic structure alterations of the ribbons induced by rehybridization were demonstrated by d*I*/d*V* spectroscopy.

## Methods

### Sample Preparation

All synthetic procedures and data acquisition were performed in two separate ultra-high-vacuum chambers (STM and HREELS) with base pressures below 1 × 10^–10^ mbar and equipped with surface preparation and analysis facilities. The precursor 10,10′-dibromo-9,9′-bianthryl (DBBA) was used to prepare the 7-AGNRs on a clean Au(111) surface.^12^ The 7-AGNRs were hydrogenated by leaking molecular hydrogen through a home-built molecular cracker consisting of a Swagelok pipe to which a glass bulb was fitted, terminating in a nozzle. A tungsten filament housed in the glass bulb was brought to incandescence to dissociate the hydrogen gas (we estimate 1% ratio H/H_2_ from^40^). During hydrogen exposure, the Au(111) sample was maintained at low temperatures (223 K for STM measurements, and 263 K for HREELS measurements), and the exposure time (hydrogen partial pressure) was set to either 200 s (1 × 10^–6^ mbar), 300 s (2 × 10^–6^ mbar), or 900 s (5 × 10^–6^ mbar), achieving doses of 200, 600, and 4500 Langmuir, respectively. Then the sample was annealed at 473 K for 200 s to remove hydrogen atoms weakly adsorbed on the Au(111) surface. To verify that no H desorption from the GNRs occurs during this conditioning stage, tests were conducted whereby the sample was further annealed to higher temperatures. Structural differences that can be ascribed to dehydrogenation of the GNRs (loss of the features described in the main text) only appear from 700 K onward (data not shown), reaching complete hydrogen evolution at approximately 780 K.

### Scanning Tunneling Microscopy (STM)

Prior to each STM experiment, the Au(111) crystal was cleaned by repeated cycles of Ar^+^ sputtering at 1 kV for 7 min and annealing in a vacuum to 823 K for 15 min. After preparation, the sample was transferred to the STM chamber. The measurements were acquired at low temperature (∼5 K), in constant-current mode with the stated voltage referring to the potential applied to the sample with respect to the STM tip. A CO-functionalized tip was used to obtain high-resolution images. Carbon monoxide (CO) was deposited on Au(111) with manipulator head cooled by LHe. A bias (−40 mV) was applied to the tungsten tip to pick up a CO molecule and form the functionalized tip. The vibrational fingerprint of these features was acquired by averaging 20 d*I*/d*V* spectra on each feature to enhance the signal-to-noise ratio, and then the result was numerically differentiated to obtain d^2^*I*/d*V*^2^ spectra. Modulation amplitudes for the lock-in amplifier detection are reported in the figure captions (and refer to peak-to-peak voltages). The BRSTM images were obtained using a CO-functionalized tip by acquiring capacitive images (d*I*/d*V* imaging with lock-in amplifier set out-of-phase) with typical parameters: *V*_*s*_ = 80 mV, *I* = 70 pA, *f* = 427 Hz, *V*_ac_ = 30 mV, *T* = 4.5 K).

We note here that it is customary to record d^2^*I*/d*V*^2^ measurements symmetric to bias polarity for the unambiguous assignment of IET vibrational resonances, in the absence of any other vibrational spectroscopy support. We consider, however, the agreement of our macroscopic vibrational data (HREELS) and single-molecule data (d^2^*I*/d*V*^2^-STM) to be undisputable for a proper assignment of our spectral features. As such, we have not performed measurements symmetric to bias polarity.

To minimize the occurrence of dehydrogenation events, which would invalidate the interpretation of the spectroscopic data, mild spectroscopy parameters (*V*_s_ = 0.5 and *I* = 0.02 nA) are used to acquire the spectra. Since the tunneling current is low and therefore the spectroscopy d*I*/d*V* signal is noisy, spectra are acquired over tens of identical features, and averaged to increase the signal-to-noise ratio. In doing so, not all the averaged spectra are acquired with the same STM tip and on the same day. However, with careful conditioning of the STM tips (via gentle controlled crashes into the Au surface and mild voltage pulses), the spectra are resilient to different tip states. This can be seen in when comparing Figure 8 (averaged spectra from different tips on different days) to Figure S11 (spectra acquired on features with the same tip).

The STM-tip assisted dehydrogenation procedures consisted in positioning the tip above hydrogenated features with *I* = 50 pA and *V* = 1 V. These tunneling parameters lead to no apparent interaction with the nanoribbons. The voltage was then ramped to 3 V over 15 s to force the removal of hydrogen from the nanoribbons by electron stimulated desorption.^41^ To ascertain the reversibility of the hydrogenation mechanism, STM images were recorded with mild image parameters after each process to show the resulting changes in electronic contrast, and to reveal the hydrogen content.

### High Resolution Electron Energy Loss Spectroscopy (HREELS)

HREELS experiments were performed in a dedicated UHV chamber with a base pressure better than 1 × 10^–10^ mbar using a VSW HIB 1000 double pass spectrometer. The spectrometer was operated in the specular geometry (θ*i* = θ*s* = 45°, where θ*i* and θ*s* are the angles formed by the incident, *i*, and scattered, *s*, electron beams with the surface normal) with primary beam energy of 6 eV and a typical elastic peak resolution of ca. 50 cm^–1^. HREEL spectra were collected after DBBA deposition on Au(111) at RT and after each annealing step (GNR synthesis). For the hydrogenation experiments, H_2_ gas was flown through a doser identical with that described in the STM section. During hydrogen exposure, the sample was held at 263 K.

### DFT Calculations

Gas phase density functional theory (DFT) calculations were performed using the B3LYP functional as implemented in Gaussian09.^42^ For comparison with HREEL spectra, geometrical optimization and calculation of the vibrational spectrum of DBBA was done using the 6-311g basis set. Energy scales were corrected according to the formula proposed by Kasahara and co-workers,^43^ to compensate for the overestimation due to the functional/basis set combination. Calculated peaks were convoluted with 50 cm^–1^ fwhm Gaussian functions. To simulate the decrease in sensitivity at increased energy loss typical of HREELS, each intensity was divided by its respective frequency to the power of 1.5.

A Gaussian 09 (RB3LYP/3-21G) procedure was first used to optimize the configuration of a sequence of pristine ribbons with increasing lengths, until the HOMO–1/LUMO+1 gap converged to 2.59 eV (Figure S10). This value is in agreement with the experimental band gap obtained from STS (dashed line). To understand the energetically preferred hydrogen docking sites, the length of 7-GNRs consisting of 10 bisanthene units with a predicted band gap of 2.65 eV was selected as an adequate model system to represent the much longer GNRs prepared experimentally. The periodic structures assumed for infinite hydrogenated ribbons were also considered and calculated with the same basis set.

For the modeling of the hydrogenation process, several hydrogenated GNRs were considered and are shown in Figure S9. Relative energies for the addition of *n* hydrogens atoms on different carbon atoms, Δ*E*_H_, were calculated by eq 1:
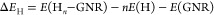
1where *E*(H_*n*_–GNR) is the total energy of the nanoribbon after the addition of *n* hydrogen atoms, *E*(GNR) is the total energy of the pristine ribbon, and *E*(H) is the total energy of a hydrogen atom.

## Supporting Information Available

HREELS measurements; BRSTM images of GNR termini; BRSTM evidence for hydrogenated GNR edge distortions; Additional STM measurements at higher H/H_2_ exposure; BRSTM image of large protrusion; STM-tip-assisted dehydrogenation measurements; DFT calculations; Electronic properties and STM tip state (PDF)

The research data supporting this publication can be accessed at https://doi.org/10.17630/881c87b3-bc04-4f53-b719-76238611f0b4.
