# Supplementary material for: Surface Confined Hydrogenation of Graphene Nanoribbons
Source: ACS Nano. 2022 Jul 5;16(7):10281–91. doi: 10.1021/acsnano.1c11372 (PMC9330764; doi:10.1021/acsnano.1c11372)
Supplement: Supplementary file 1 — nn1c11372_si_001.pdf [file nn1c11372_si_001.pdf]

Surface Confined Hydrogenation of Graphene Nanoribbons

Yi-Ying Sung, Harmina Vejayan, Christopher J. Baddeley, Neville V. Richardson,

Federico Grillo, Renald Schaub*

EaStCHEM and School of Chemistry, University of St Andrews, KY16 9ST, St Andrews, UK

Corresponding author (*): renald.schaub@st-andrews.ac.uk

Supplementary information

SI1: HREELS measurements…………………………………………………………………… Page 2

SI2: BRSTM images of GNR termini………………………………………………………… Page 3

SI3: BRSTM evidence for hydrogenated GNR edge distortions………………… Page 4

SI4: Additional STM measurements at higher H/H_2 exposure………………… Page 5

SI5 BRSTM image of large protrusion…………………………………………………… Page 5

SI6 STM-tip-assisted dehydrogenation measurements…………………………… Page 6

SI7: DFT calculations……………………………………………………………………………… Page 7

SI8: Electronic properties and STM tip state…………………………………………… Page 10

SI1: HREELS measurements

Figure S1 shows the corresponding HREEL spectra obtained from the monomer DBBA, the polymer produced after the first annealing step, and 7-AGNRs obtained after the second annealing step (cyclodehydrogenation), adsorbed on the Au(111) surface with red, blue and green traces, respectively. A background spectrum of the clean Au(111) surface is also shown as a reference (black trace). The spectra of the monomer and the polymer show several out-of-plane vibrations, such as phenyl ring torsion and out-of-plane δ(CH)oop bending modes (corresponding assignment is reported in Table S1), which arise from the tilted anthracene units. These modes record an intensity decrease after inducing the surface assisted cyclodehydrogenation to from AGNRs (green). This is because the AGNRs are essentially flat; therefore, the in-phase δ(CH)oop rocking mode at 765 cm^{-1}, which has a strong transition dipole moment perpendicular to the surface plane, is the only observed mode. On the other hand, the ν(C–H) stretch mode, expected at 2975 cm^{-1}, occurs on a plane parallel to the metal surface and therefore is not HREEL active. Note that there is also a decrease of the total amount of C–H bonds in going from the polymer to the GNRs (discounting the termini, 4 C–H bonds are broken and 2 C–C bonds are formed for every 2 DBBA condensing).

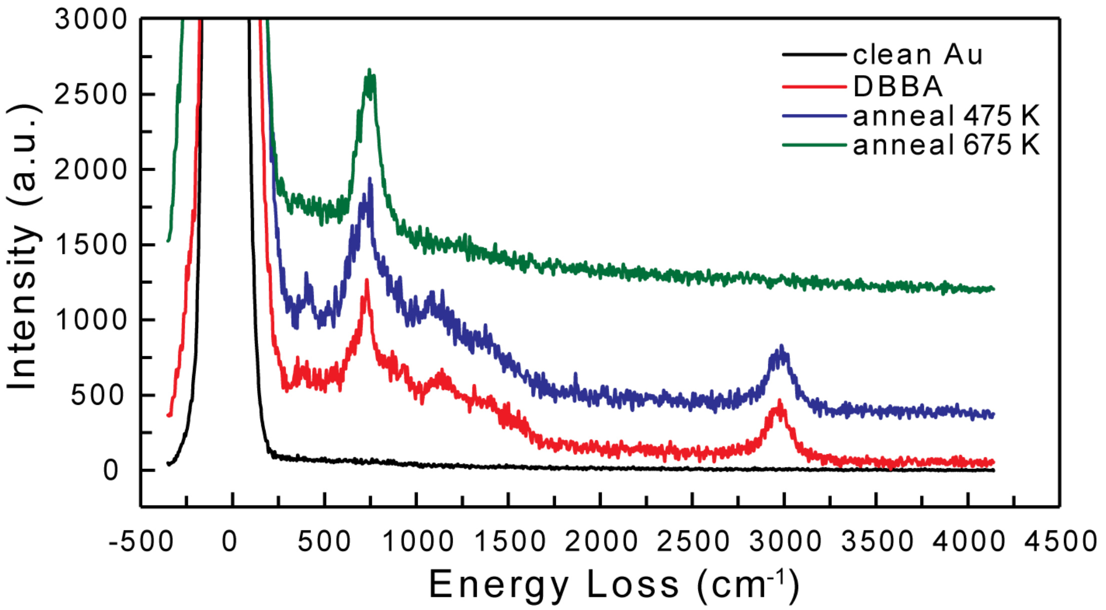

Figure S1. HREEL spectra of DBBA recorded as function of increasing annealing temperature. *Black trace records the HREEL spectrum of the clean Au(111) surface as a reference. Red trace shows the HREEL spectrum of the precursor (DBBA) on Au(111). Blue and green traces show the spectra of the polymer and the 7-AGNRs, respectively. Spectra are offset for clarity.*

Figure S2 shows a comparison between the HREEL spectrum recoded after dosing DBBA on Au(111) and the calculated gas phase vibrational spectrum of DBBA. Assignments of vibrations are reported in Table S1.

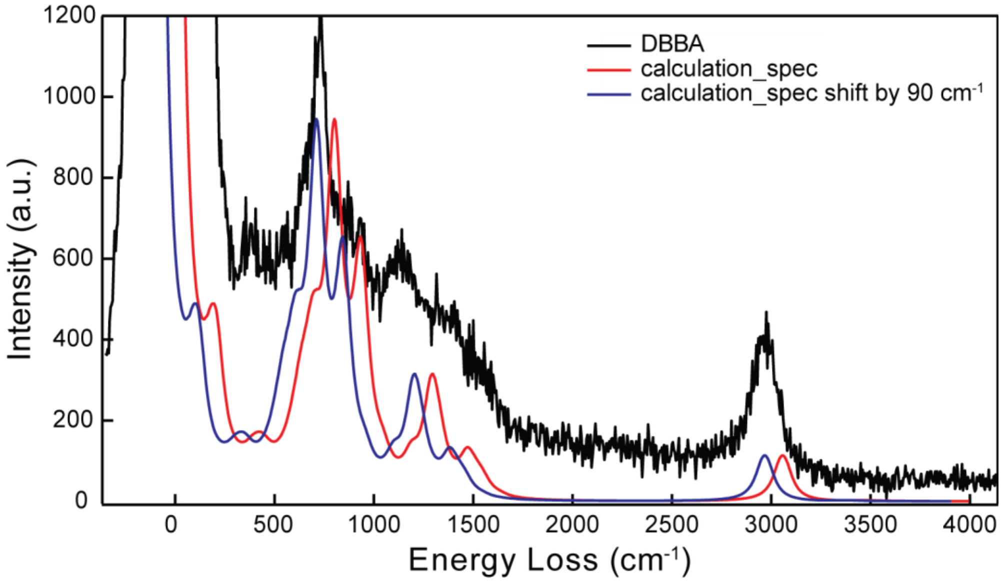

Figure S2. Comparison between the HREEL spectrum of DBBA on Au(111) and the calculated gas phase vibrational spectrum of DBBA.

Table S1: Assignment of the vibrational modes (in cm^{-1}) for DBBA

HREELS	Calculated	Mode
2965	3055	CH stretch aromatic
1575	1540	CH in plane bend
1490	1490	CH in plane bend + CC stretch (weak)
1405	1420	CC stretch
1315	1300	CH in plane bend + central rings breathing
1130	1180	CH in plane bend+ CC bend (weak)
935	930	Aromatic in plane deformation
825	800	CH out of plane bend + central rings breathing
730	705	CH out of plane bend + rings in plane bends
635	690	Rings in plane deformations
550	600	Rings twists + CH out of plane bend
460	450	Rings twists + CH out of plane bend
385	410	Rings twists + CH out of plane bend

SI2: BRSTM images of GNR termini

Figure S3 details the chemical structure of the (a) enlarged terminus (CH terminus) and (b) of the featureless terminus (CH_2 terminus). The alternation of three and two hexagonal rings corresponds to the expected 7-AGNRs structure and can be observed in the CH_2 terminus. A brighter contrast can be observed (highlighted by an arrow) at the locations of the two terminal hydrogens. In contrast, the structural details of the enlarged terminus can only be partially discerned. These results are compatible with the characterisation and interpretation of termini in refs. 36 and 37 of the main manuscript.

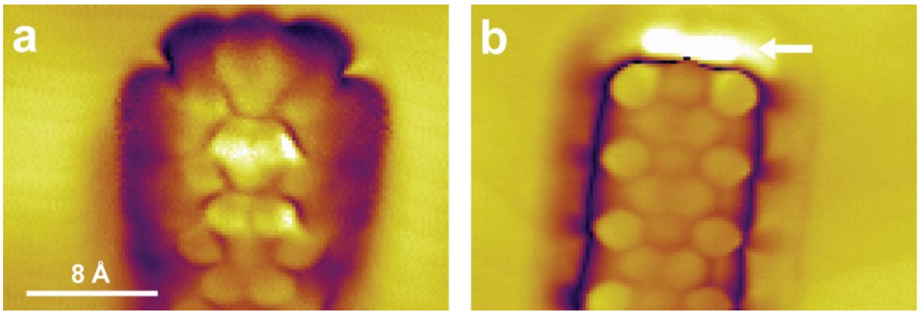

Figure S3. Intramolecular structure of 7-AGNR termini in BRSTM images. *(a) Enlarged CH terminus, (b) Featureless CH_2 terminus. Tunnelling parameters: (a) V_s = 40 mV, I = 0.8 nA, f = 427 Hz, V_{ac} = 40 mV; (b) V_s = 40 mV, I = 0.8 nA, f = 427 Hz, V_{ac} = 40 mV.*

SI3: BRSTM evidence for hydrogenated GNR edge distortions

To emphasise the subtle asymmetric appearance of the edge C_6 rings directly affected by the hydrogenation shown in Figure 4g, we have subjected the BRSTM to an edge-enhancing Laplace filter as shown in Figure S4.

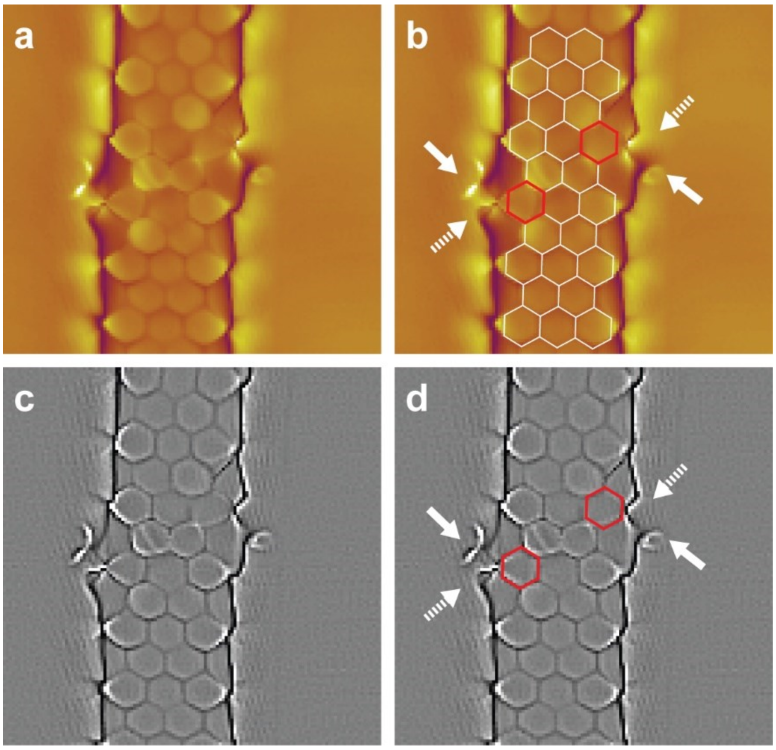

Figure S4 BRSTM and edge-enhancing Laplace filtered BRSTM image of a small edge defect. *The BRSTM image shown in both panels (a) and (b) is identical to the one reported in Figure 4g of the main manuscript (see Figure 4 caption for details). Panels (c) and (d) show the result of applying an edge-enhancing Laplace filter to highlight the asymmetry observed at the edge defects. Note in particular the asymmetric occurrence (with reference to the hydrogenated C6 ring marked in red) of bright features on either side of the ribbon indicated by the solid arrows.*

SI4: Additional STM measurements at higher H/H_2 exposure

A series of STM images with two hydrogenation conditions (condition 1: 600 L; condition 2: 4500 L) is analysed by applying two binary threshold operations to discriminate both the regions ascribed to ribbons and to the protrusions from the background. This allows to provide a crude estimate of the fraction of the hydrogenated AGNRs. Figures S5a and S5b show two examples of the resulting analysis (coloured delineation of the binary boundaries) superimposed on the STM images corresponding to Figures 5d and 5e of the main text, respectively. The white lines highlight regions of 7-AGNRs, and the blue lines mark the protrusions. The ratio of areas indicates approximately 39% of hydrogenated nanoribbon sections for procedure 1 and 32% for procedure 2 (analysis is based on several STM images). Although our results merely show a rough estimate of the percentage of the protrusions as compared to the nanoribbons, these indicate that the hydrogenation process is self-limited.

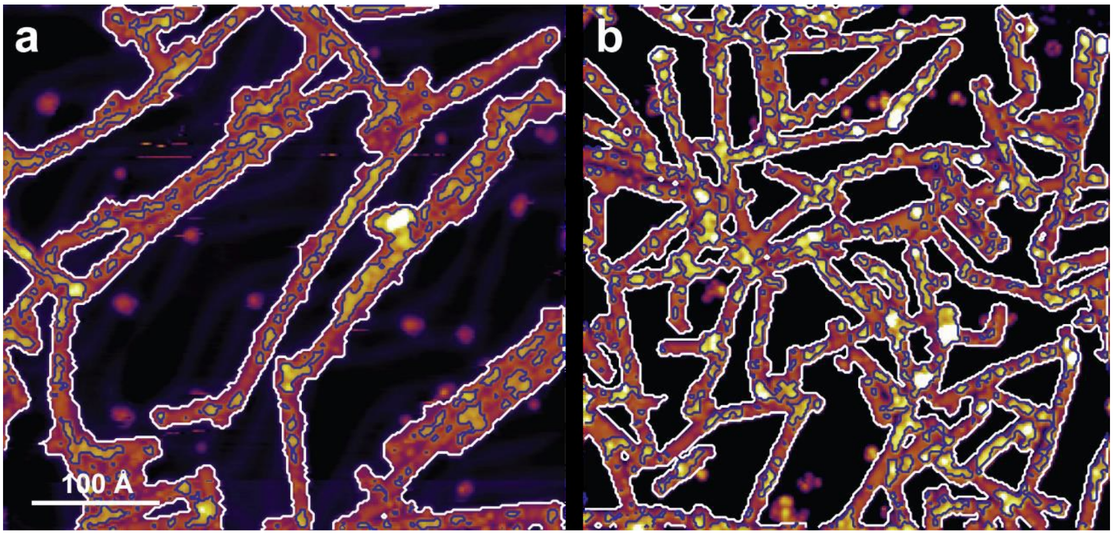

Figure S5 Examples of binary threshold analysis of hydrogenated features. *Binary images are obtained by binary thresholding the images show in Figure 5d, e with reference to the apparent height of the ribbons and the hydrogenated features. The regions of the ribbons are highlighted in white, and the protrusions are marked in blue.*

SI5 BRSTM image of large protrusion

Figure S6 presents the case of a large protrusion. Figure S6b shows the corresponding BRSTM image of the same nanoribbon as in Figure S6a. Since the nanoribbon is more distorted, the BRSTM image does not reveal the intramolecular structure of the large protrusion. Nevertheless, the superposed model (Figure S6c) illustrates the extent of the distorted section and suggests that further hydrogenation occurs on the basal plane rather than at the edge. Note that the asymmetric edge appearance is caused by an asymmetric CO-functionalised tip.

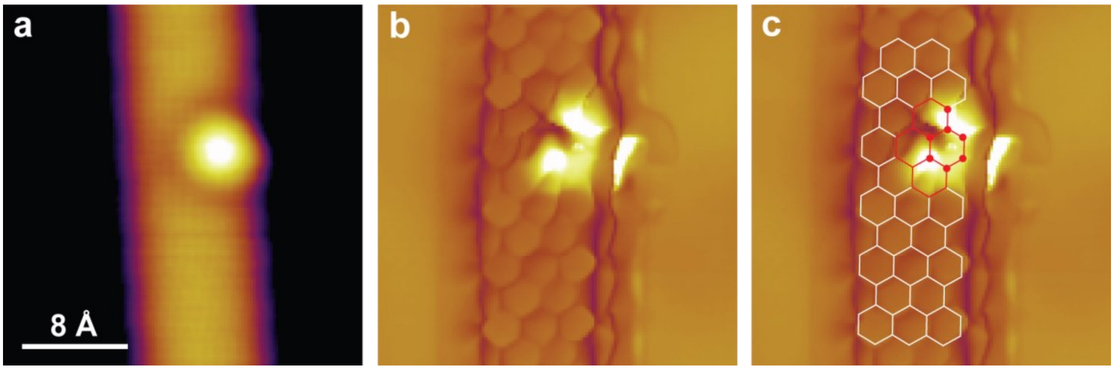

Figure S6 Imaging of a large protrusion at an edge of AGNR. *(a) STM topographic image and (b) BRSTM image of a large protrusion identified on 7-AGNR. (c) The superposed model highlights the electronic contrast associated with the larger protrusion. Tunnelling parameters: (a)* V_s *= 1.0 V, I = 0.1 nA; (b)* V_s *= 40 mV, I = 1.0 nA, f = 427 Hz,* V_{ac} *= 60 mV.*

SI6 STM-tip-assisted dehydrogenation measurements

Figure S7a shows a high magnification STM image of an intermediate feature ascribed to a configuration consisting of an odd number of edge hydrogen atoms (3 in total, consisting of 2 from the pristine ribbon and 1 added). An asymmetric contrast can be observed at the position of the feature. Figure S7b shows the corresponding BRSTM image. Most of the hexagon rings can be distinctly discerned. A superimposed model highlights that only one distorted hexagon ring marked with a red hexagon (Figure S7c) is involved in the structure of the intermediate feature. It displays a notably high and asymmetric electronic contrast confined to the edge of the nanoribbon.

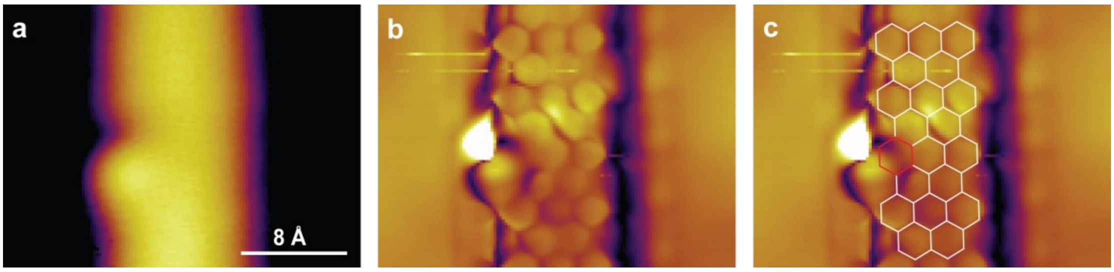

Figure S7. Intramolecular structure of the intermediate feature. *(a) Standard STM image, and (b) BRSTM image. (c) The superposed model highlights the electronic contrast associated with the intermediate feature. Tunnelling parameters: (a)* V_s *= 1.0 V, I = 0.1 nA; (b)* V_s *= 40 mV, I = 1.0 nA, f = 427 Hz, Vac = 60 mV.*

A tip-assisted dehydrogenation procedure is applied to a large edge defect (Figure S8a). STM images acquired after each manipulation step are shown in Figure S8b, S8c and S8d. These reveal a sequence evolving from the large edge defect to an intermediate feature (with an odd number of H atoms, consisting of 2 from the pristine ribbon and 3 added), a small edge defect, and finally a pristine nanoribbon, respectively.

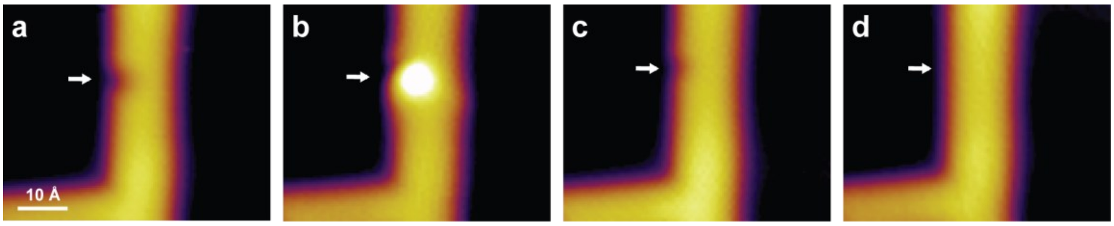

Figure S8 Stepwise tip-induced dehydrogenation of a large edge defect. *STM image of (a) a large edge defect, (b) an intermediate feature, (c) a small edge defect, and (d) a featureless site. The arrows highlight the exact dehydrogenation sites. Tunnelling parameters: V_s=0.5 V, I=0.05 nA.*

A further example of tip-assisted dehydrogenation, together with corresponding d*I*/d*V* spectra is reported below, in Section SI8.

SI7: DFT calculations

As described in the main manuscript, we performed DFT calculations on two different model systems of H_2-AGNR to study theoretically the hydrogenation process (Figure S9):

System #1: A finite 7-AGNR composed of 10 anthracene units with CH_2 termini and with a hydrogenated C-C pair at a central anthracene unit (6th unit from one side, 5th from the other). The minimum length of the GNRs was determined by evaluating the convergence of the HOMO/LUMO and HOMO-1/LUMO+1 gaps [L. Talirz *et al.*, ChemPhysChem 2019, **20**, 2348-2353] with increasing number of anthracene units, as in Figure S10. From this, we observe convergence of the gaps for 10 anthracene units.

System #2: An infinite ribbon with a hydrogenated C-C pair repeated after 4 unit cells – a separation that is sufficient to minimise the physical interaction between adjacent hydrogenated C-C pairs. For sake of completeness, we also report an infinite ribbon with a hydrogenated C-C pair repeated after 2 unit cells – a separation whereby the close proximity of C-C pairs affects the calculation results, as shown in Tables S2, S3.

In Table S2, the calculation results show that the tilted hydrogenated C-C pair is more stable than a flat configuration and possesses a similar local geometry to calculations reported in ref. 34 of the main manuscript on the hydrogenation of 29-AGNRs. As a result, all the model systems considered here have tilted hydrogenated C-C pairs, where one of the edge hydrogenated carbon moves upward with respect to the plane of the nanoribbon (filled red circle) and the other one moves downward (open red circle). As described in the main manuscript, our results here agree with previous calculations.

From this point on, we proceed to investigate theoretically the addition of a further H atom ((H_2+H)-AGNR) in view to understand how the hydrogenation mechanism extends onto the ribbon from

the tilted hydrogenated C-C edge pair. We considered 8 possible adsorption sites in vicinity of the C-C edge pair: 4 pairs of two sites symmetrically located on either side of the C-C edge pair. These are colour-coded in Figure S9 by yellow circles on C1 edge sites, green and blue circles on C2 basal plane sites, and purple circles on C3 basal plane sites. Due to the tilted edge structure, the H_2-AGNRs no longer possess D_{2h} symmetry. Consequently, the two symmetric sites of each pair are rendered energetically inequivalent for adsorption and are differentiated by open and filled circles in Figure S9. Table S3 shows the relative energy computed at each site. In the lowest relative energy configuration, the third hydrogen atom prefers to bind to a C2 basal-plane site in direct vicinity of the hydrogenated C-C edge pair (blue open circle) rather than to edge carbon atoms (both yellow circles on either side of the C-C pair). Therefore, from an initial small edge defect (whose formation involves only edge hydrogenation *via* a pairwise addition mechanism), our results indicate that further hydrogenation will proceed onto basal plane sites, therefore providing support to limitations in lateral extension for the hydrogenation reaction. This theoretical prediction is consistent with our experimental observations. For there on, we hypothesize that two competing reaction pathways can lead to either the formation of a large edge defect or a small protrusion as discussed in our main manuscript.

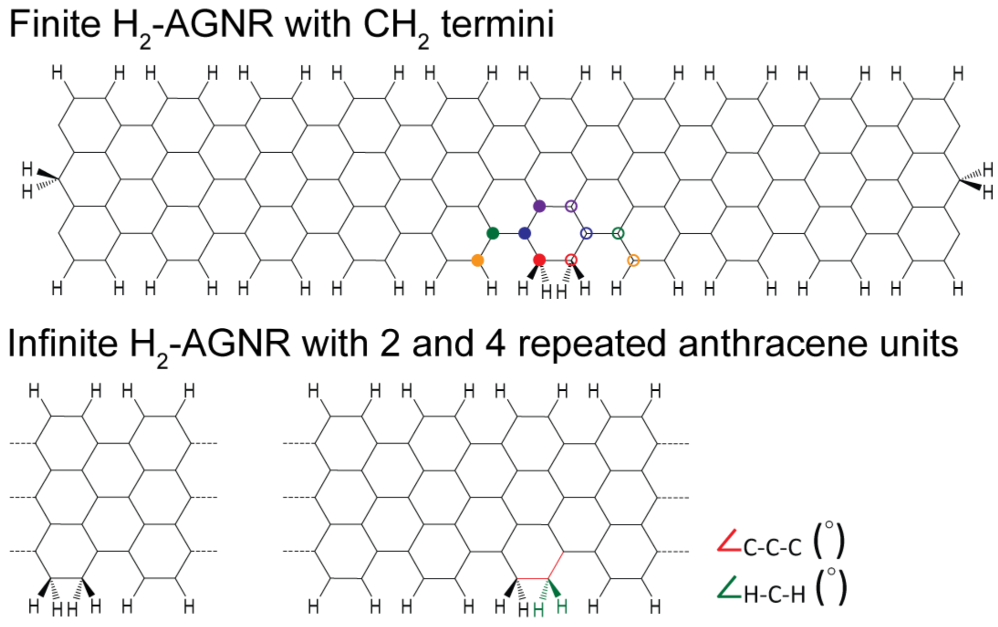

Figure S9 The structures of the two model systems used for modelling the hydrogenation process. *The bond angle$\angle_{C\text{-}C\text{-}C}$ is highlighted in red, and $\angle_{H\text{-}C\text{-}H}$ is highlighted in green. The red circles highlight the edge hydrogenated carbon atoms. To indicate the tilted edge structure, the open circles indicate carbon atoms relaxing at positions lying below the plane of the pristine ribbon, and the filled circles indicate carbon atoms relaxing at positions above the plane. We considered six possibilities of hydrogenated sites on the basal plane highlighted in colour dots (green, blue and purple), and two possible edge hydrogenated sites highlighted in yellow.*

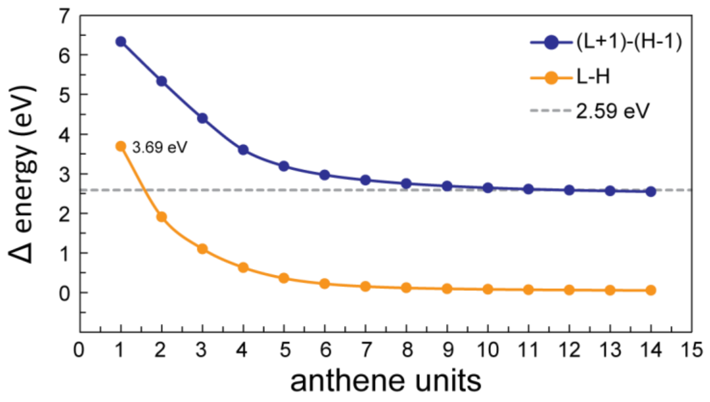

Figure S10. Variation of HOMO/LUMO and HOMO-1/LUMO+1 gaps with increasing number of anthracene units.

Table S2. The relative adsorption energy of pairwise hydrogenation (ΔE_H), the energy differences (ΔE_f) relative to the flat edge structure, the bond angles ($\angle_{C\text{-}C\text{-}C}$ and $\angle_{H\text{-}C\text{-}C}$) and the bond lengths ($d_{C\text{-}C}$) of the hydrogenated C-C bonds are listed for the two model systems of H_2-AGNRs with tilted edge structure, and compared with calculation results from Choe et al. (ref. 34 in the main manuscript).

	10 units		2 repeated units		4 repeated units		ref. 34	
	flat	tilted	flat	tilted	flat	tilted	flat	tilted
ΔE_H (eV)	-3.047	-3.176	-5.486	-5.592	-5.471	-5.596	-	-
ΔE_f (eV)	0	-0.129	0	-0.106	0	-0.125	0	-0.060
$\angle_{C\text{-}C\text{-}C}$ (°)	116.7	110.9	116.8	111.5	116.7	111.2	117.0	111.8
$\angle_{H\text{-}C\text{-}H}$ (°)	106.7	107.8	106.7	107.6	106.7	107.8	104.0	106.3
$d_{C\text{-}C}$ (Å)	1.546	1.533	1.545	1.531	1.545	1.533	1.519	1.511

Table S3 The relative adsorption energy of a third hydrogen atom on different carbon sites, which are indicated by their corresponding coloured dots in Figure S9.

Unit: eV	10 units	2 repeated units	4 repeated units
Case I-up (Blue filled circle)	-1.665	-1.967	-1.627
Case II-up (Green filled circle)	-0.697	-0.886	-0.752
Case III-up (Purple filled circle)	-1.071	-1.009	-1.045
**Case I-down (Blue open circle)**	**-1.970**	**-1.967**	**-1.978**
Case II-down (Green open circle)	-0.695	-0.758	-0.688
Case III-down (Purple open circle)	-1.071	-1.036	-1.067
Edge-up (Yellow filled circle)	-1.713	-1.667	-1.715
Edge-down (Yellow open circle)	-1.713	-1.667	-1.715

SI8: Electronic properties and STM tip state

Figure S11a shows a large edge defect. Subsequent tip-assisted dehydrogenation manipulations were performed and the corresponding STM images are reported in Figures S11b, S11c and S11d. These exhibit a small edge defect (b), an intermediate feature (c) and a pristine nanoribbon (d). Constant-height d*I*/d*V* spectra recorded at each feature (the exact acquisition positions are highlighted with coloured dots) with the same STM tip are shown in Figure S11e. The spectra confirm the negligible influence of the STM tip state (compare Figure S11e to Figure 8). In addition, dehydrogenated AGNRs shows nearly identical electron structure as compared to pristine AGNRs indicating that the adsorbed hydrogen atoms can be fully desorbed by using tip-induced dehydrogenation without forming any defect.

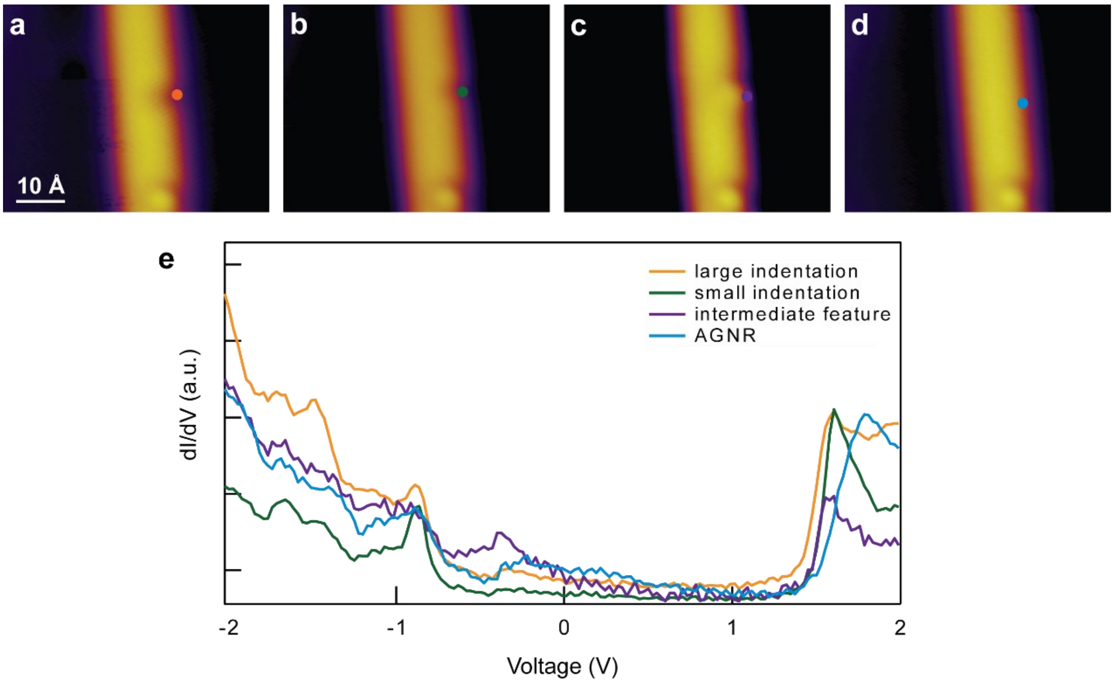

Figure S11 Electronic structure of a large edge defect, a small edge defect, an intermediate feature and the pristine AGNR. *Representative STM topographic image of (a) a large edge defect, (b) a small edge defect, (c) an intermediate feature, and (d) a AGNR after manipulation. Colour dots marked the location of acquisition of the dI/dV spectra over the features. (e) STM dI/dV point spectra taken at the locations indicated with dots in the STM images. Tunnelling parameters:* $V_s = 0.5$ *V,* $I = 0.1$ *nA. Spectroscopy parameters:* $V_s = 0.5$ *V,* $I = 0.02$ *nA,* $f = 423$ *Hz,* $V_{ac} = 40$ *mV.*
